# The association between Trichomonas tenax and Entamoeba gingivalis and periimplantitis and periodontitis

**DOI:** 10.1007/s00784-026-07036-x

**Published:** 2026-07-17

**Authors:** Aysegul Sari, Ozlem Makbule Kaya, Volkan Arisan, Luigi Nibali

**Affiliations:** 1 Periodontology Unit, King’s College London Dental Institute, Centre for Host-Microbiome Interactions, London, UK; 2 Faculty of Dentistry, Department of Periodontology, Hatay Mustafa Kemal University, Hatay, Turkey; 3 Faculty of Dentistry, Department of Parasitology, Hatay Mustafa Kemal University, Hatay, Turkey; 4 Faculty of Dentistry, Department of Oral Implantology, Istanbul University, Istanbul, Turkey

**Keywords:** Periimplantitis, Periodontitis, Trichomonas tenax, Entamoeba gingivalis

## Abstract

**Objective:**

The aim of the study was to evaluate the association between *T. tenax* and *E. gingivalis* with peri-implant and periodontal diseases compared to healthy controls.

**Material and Methods:**

A total of 140 participants were included in four groups in this study. The groups included 35 patients with peri-implantitis and periodontitis (IP+P group); 35 patients with peri-implant and periodontal health (IH+PH group); 35 patients with periodontitis (P group), and 35 periodontally healthy individuals (PH group). Clinical periodontal and peri-implantitis parameters were recorded. Plaque samples were taken from around teeth in P and PH groups and around implants and teeth in IP+P and IH+PH groups. The presence of *T. tenax*and*E. gingivalis* was determined under a microscope.

**Results:**

The detection of *T. tenax* and *E. gingivalis* parasites in tooth samples was higher in the IP+P and P groups than in the IH+PH and PH groups (*p*<0.001). Both parasites were detected more frequently in peri-implant samples in the IP+P group than in the IH+PH group (*p*<0.001 and *p*=0.001, respectively). There was no difference in parasite detection between tooth and peri-implant samples in the same host in the IP+P and IH+PH groups (p³0.05). The presence of *T. tenax* and*E. gingivalis *was associated with the presence of periodontal disease, and severity and grading of periodontitis independently of other factors (*p*<0.05).

**Conclusion:**

The presence of *T. tenax* and *E. gingivalis* might be associated with peri-implantitis and periodontitis lesions. The detection of parasites is similar on tooth and implant surfaces in the same mouth.

**Clinical Relevance:**

Protozoa found at sites of disease may not imply a direct causal relationship with the disease; nevertheless, their detection can indicate opportunistic microbial activity or ongoing oral inflammation.

**Supplementary Information:**

The online version contains supplementary material available at 10.1007/s00784-026-07036-x.

## Introduction

Peri-implantitis is characterized by infection and tissue loss in the supporting tissues of dental implants as a result of peri-implant biofilm accumulation [1]. Estimates of the prevalence of peri-implantitis at the patient level range from 22 to 19.83% [2, 3]. Clinically, increased probing depth, mucosal recessions, inflammation, and bleeding on probing accompany radiographic bone loss in the diagnosis of peri-implantitis [1].

Peri-implant plaque accumulation is the main etiological factor that plays a role in the initiation and progression of peri-implantitis [4]. Disruption of tissue homeostasis and microbial dysbiosis and the host can lead to peri-implant diseases [5]. The studies show that the microbial appendage causing peri-implantitis is heterogeneous and complex [6], including opportunistic pathogens such as *Pseudomonas aeruginosa* and *Staphylococcus aureus* [7]; fungal organisms such as Paelicomyces spp, Penicillum spp, and *Rhadotorula laryngis* [8]; and viral organisms such as Epstein Barr virus and Cytomegalovirus [9]. In recent years, advanced meta-genomic and transcriptomic studies highlighted the importance of the presence of other members of the microenvironment such as viruses, fungi, and archaea in the microbial community of periodontal and peri-implant diseases [10].

In the 1980 s, eukaryotes were suggested to play a role in this hysterogenesis and complex structure [11, 12]. *Entamoeba gingivalis* (*E. gingivalis*) and *Trichomonas tenax* (*T. tenax*) are the two most common parasites in dental plaque [13, 14]. The presence of *E.gingivalis* in infected gingival tissue was confirmed by polymerase chain reaction. Microscopic examinations showed that the amoeba moved within the gingival tissue, penetrated the cytoplasm of viable host gingival epithelial cells, and took fragments from the nuclei of host cells [15]. *E. gingivalis* was visualized in the mucosa surrounded by abundant neutrophils in gingival tissue with gingival inflammation [15]. It was previously suggested that neutrophils would probably not resolve *E. gingivalis* infection [16], leading to increased neutrophil invasion, exacerbation of periodontal inflammation, and tissue destruction [15]. Although the prevalence of *E. gingivalis* is at undetectable levels in healthy periodontium, its high prevalence can be seen in periodontal disease, ranging from 30 to 80% [17]. The incidence of *T. tenax*, a single-cell anaerobic motile-flagellated protozoan in oral cavity, ranges from 0% to 94.1%, depending on the region and the detection procedure [18]. It has also been reported that *T. tenax* disrupted cell junctions, causing a cytotoxic effect on gingival epithelial cells and induced IL-6 production in the presence of low-dose infection [19].

In recent years, there have been studies focusing on differences between peri-implant and periodontal site microbial communities, even if the differences have not been conclusively established [20, 21]. Microbial migration from subgingival tooth sites to peri-implant niches may occur [22]. Microbial profiles vary between healthy teeth, periodontitis, and peri-implantitis sites and it is highlighted that the qualitative aspect of the microbial composition is an important disease risk factor [6]. In most previous studies, diseased and healthy sites were sampled from different individuals [23, 24], while very few studies sampled them from the same individuals [20, 21].

To the best of the authors’ knowledge, there is only one study in the literature investigating the relationship between *E. gingivalis* and *T. tenax* parasites and periodontitis. Also, no study is comparing the prevalence of both parasites in the peri-implantitis and periodontitis microbiological niches. Therefore, there is a need to analyze plaque samples from both implant and tooth sites from the same subject. In the present cross-sectional study we hypothesized that there is an association between *T. tenax* and *E. gingivalis* and peri-implant and periodontal diseases compared to healthy controls, and also that these parasites are differently detected in sites of implant and tooth in the same patients.

## Materials and methods

### Study population

This cross-sectional study was carried out in the Department of Periodontology in the Faculty of Dentistry and the Department of Parasitology in the Faculty of Medicine at Hatay Mustafa Kemal University, Hatay, Turkey. The study protocol was approved by the Ethics Committee for the Use of Human Subjects in Research of Hatay Mustafa Kemal University, Hatay, Turkey (Protocol No: 2020/35) and the study was carried out in accordance with the tenets of the Declaration of Helsinki. Strengthening the Reporting of Observational Studies in Epidemiology (STROBE) guidelines for observational studies were followed [25].

This study included 140 patients divided into four groups: peri-implantitis and periodontitis (IP + P: test group) (35 patients); peri-implant and periodontal health (IH + PH: control group) (35 patients); periodontitis (P: control group) (35 patients) and periodontal healthy controls (PH: control group) (35 participants). The patients were recruited from the Department of Periodontology, Faculty of Dentistry at Hatay Mustafa Kemal University, from January 2020 to January 2022. Dental faculty students and staff were included as periodontal healthy controls. The study protocol was explained, and written informed consent was received from each individual prior to enrolment.

The inclusion criteria for study groups were: (i) age ≥18 years old and (ii) body mass index (BMI) ≤30 kg/m for all groups. Also, the inclusion criteria for patients with implants were: (i) at least 2 years after implant placement, (ii) at least 1 year after prosthesis placement, (iii) at least 1 implant diagnosed with peri-implantitis and overall diagnosis of periodontitis (IP + P group) or at least 1 healthy implant and overall healthy periodontal conditions (IH + PH group), (iv) implant inserted in native bone with no guided bone regeneration. The prosthetic design of the included implant units consisted of either single crowns or fixed partial dental prostheses.

Exclusion criteria for study groups were patients who had undergone antiprotozoal, antibiotic, or periodontal therapy in the past 6 months, and pregnancy and/or hormonal and/or lactation therapy. Also, patients with fully edentulous or full-mouth implants were excluded from the study.

After explaining the study protocol and obtaining formal written consent from individuals, demographic variables, smoking status, systemic disease status, tooth brushing frequency, and frequency of dental check-ups were collected.

### Peri-implant and periodontal examination

Upon confirmation of eligibility for enrolment in the study full-mouth and sampling, clinical periodontal parameters including plaque index (PI) [26], gingival index (GI) [27], bleeding on probing (BOP) (presence/absence) (%) [28], probing pocket depth (PPD) (mm), and clinical attachment loss (CAL) (mm) were recorded from all participants during their visit to the Periodontology Department. Clinical periodontal measurements were performed at six sites on each tooth (mesio-buccal, mid-buccal, disto-buccal, mesio-lingual, mid-lingual, and disto-lingual locations), except for third molars, using a manual periodontal probe (Williams, Hu-Friedy, Chicago, IL, USA) by a single, previously calibrated examiner (A.S.). Mean probing pocket depth and clinical attachment loss were also calculated. Intra-examiner agreement was determined for CAL by using the Intraclass Correlation Coefficient (ICC). The intra-examiner reproducibility was determined through repeated examinations of 10 subjects with a one-hour interval (ICC = 0.91).

Radiographic examination was performed for all groups. Marginal peri-implant and periodontal bone loss was evaluated on standardized long-cone periapical radiographs obtained with a parallel technique using Emago software (Oral Diagnostic Systems, Amsterdam, The Netherlands). The linear radiographic analysis was carried out by a single calibrated examiner (AS). The marginal bone level was measured from the implant shoulder and/or tooth cemento-enamel junction to the initial bone-implant contact.

Periodontal and peri-implant diseases were diagnosed according to clinical and radiographic criteria proposed by 2017 World Workshop on the Classifications of Periodontal and Peri-implant Disease and Conditions [29]. Individuals with BOP<10% without attachment loss and radiographic bone loss were considered to have periodontal health [30]. The criteria for patients with periodontitis were interdental CAL detectable at ≥ 2 non-adjacent teeth or buccal or oral CAL ≥ 3 mm with PPD > 3 mm detectable at ≥ 2 teeth. Peri-implant health was determined by an absence of visual signs of inflammation, bleeding on probing, and further bone loss following initial healing, which should not be ≥ 2 mm. Therefore, no implants with bone loss beyond initial remodeling were included in the peri-implant health group. The criteria for patients with peri-implantitis were PPD ≥ 6 mm in conjunction with profuse bleeding and ≥ 3 mm bone loss from the implant shoulder [1, 31]. The categorized greatest values of interdental CAL, which is defined as the distance from the cemento-enamel junction to the base of the periodontal pocket, for periodontitis severity were defined according to the periodontitis stages [32]. Mild periodontitis was defined as the presence of CAL: 1 to 2 mm (Stage I); moderate periodontitis was defined as the presence of CAL: 3 to 4 mm (stage II); severe periodontitis was defined as the presence of CAL ≥ 5 mm (stage III) and the presence of CAL ≥ 5 mm with ≥ 5 tooth loss due to periodontitis and/or advanced complexity (stage IV) [32]. Grading was assigned based on bone loss/age ratio and modifying factors (smoking or metabolic control of diabetes) [33].

### Samples collection

**Fig. 1:**
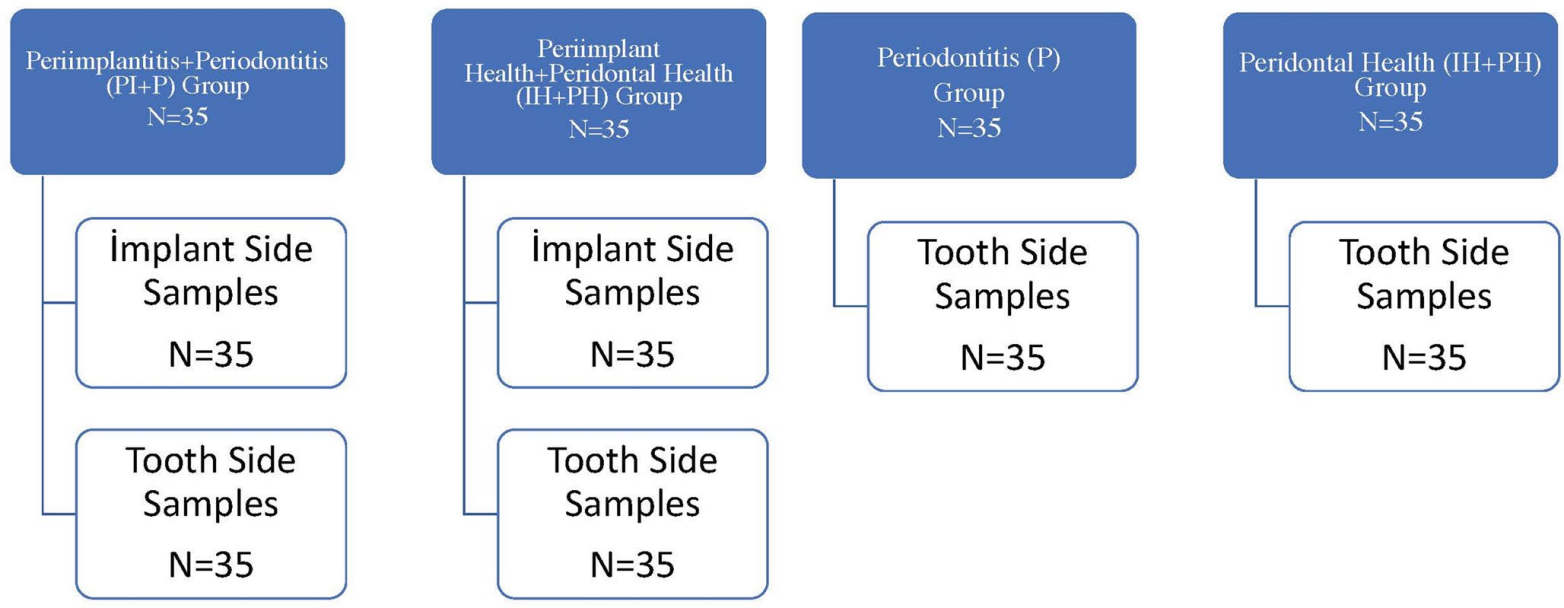
The diagram with the 4 groups and the sampled

Plaque samples were taken from implants (70 samples) and teeth (70 samples) in the PI + P and IH + PH groups, and from teeth only in the P (35 samples) and PH groups (35 samples) (Fig. 1) using a sterile Gracey curette (HuFriedy, Rotterdam, Netherlands). Samples were collected from single teeth and implants that were not adjacent to each other in the periimplantitis groups. Subgingival implant and dental plaque samples and gingival crevicular fluid (GCF) and peri-implant crevicular fluid (PICF) were collected from the two deepest peri-implant and periodontal pockets by inserting a standard paper strip (Periopaper; Oraflow Inc., Plainview, NY) within 10 s in PI + P and P groups. GCF or PICF and plaque samples were combined in the same eppendorf (Araujo & Lindhe, 2018). The same method was used for healthy gingival sites in the IH + PH and PH groups. The sampling sites were isolated from saliva and slightly air-dried. The sample was collected using a sterile Gracey curette (HuFriedy, Rotterdam, Netherlands) and transported into 1 mL of %0.9 NaCl solution.

### Microscopic examination

Samples were immediately delivered to the parasitology laboratory. Two slides were prepared for each sample. The detection of parasites was performed by direct microscopy [34] and the Giemsa staining method [35] under microscopic examination (Olympus CH20; Olympus Optical, Tokyo, Japan) by a medical parasitology specialist (O.M.K). The first method was a direct microscopic examination. Microscopic examination was performed at 100x and 400x magnification. The samples were spread on the slides using a pasteur pipette, dried in air, and fixed chemically for approximately 1 min with methanol (Merck 1.06009). In the second method, the samples were stained with Giemsa dye solution (Merck 1.09204; Giemsa’s azure–eosin–methylene blue) for 30 min and examined under a light microscope at 1000× magnification using oil immersion. Representative microscopic images of *E. gingivalis* detected in the examined samples are shown in Supplementary Fig. 1. To ensure standardization, each slide was systematically examined across the entire smear area using a uniform scanning protocol with non-overlapping fields. The method-level positivity for each sample (direct+/Giemsa+, direct+/Giemsa−, direct−/Giemsa+, direct−/Giemsa−) was determined. A sample was classified as negative only when no parasitic forms were detected in either preparation following complete microscopic screening of the slide. Laboratory personnel involved in parasite detection were blind to the clinical origin of the samples.

### Statistical analysis/Power calculation

The primary outcome of this study was the detection of parasites in peri-implant and periodontal diseases compared to healthy controls. The secondary outcome of this study was the detection of the parasites in peri-implant and periodontal plaque in the same host. A separate unpublished pilot analysis regarding the sample size was performed, including 12 subjects in two of the groups (peri-implantitis vs. peri-implant health) to determine the effect size of the study for the presence of *E. gingivalis*.

The minimum required sample size was established to be 28 for each group to detect a significant difference in the presence of *E. gingivalis* between the four groups (effect size = 0.53, 90% power, and 95% confidence). The normality of the distribution of continuous variables was examined by Shapiro-Wilk test. Mean ± standard deviations (mean ± SD), median, 1 st quartile, and 3rd quartile for numerical variables, and frequencies and percentages for categorical variables were given as descriptive statistics. Comparing two groups for non-normally distributed variables was examined by performing Mann-Whitney U test. More than two non-normally distributed variables were compared by performing Kruskal-Wallis test and post hoc pairwise multiple comparison analyses were performed by Dunn multiple comparison test. Associations between categorical variables were tested using Chi-squared tests; p values were corrected according to Bonferroni.

The effect of independent variables in the presence of *T.tenax* and *E. gingivalis* was determined by univariate and multiple logistic regression analysis. Variance inflation factors (VIFs) were calculated to assess the multi-collinearity problem, only variables with VIF < 5 [36] were included in the multiple models. Multivariate logistic regression analysis was performed by the Backward method. The models were used to estimate associations between the presence of parasites (exposures) and periodontal and peri-implant disease parameters (outcomes). The different multiple model analyses were performed for each outcome variable (periodontal group, periodontitis severity, and periodontal progression rate) until they reached the final models. Covariates used for for model analysis were age, sex, BMI, smoking status, systemic health status, frequency of tooth brushing, and frequency of dental check-ups. The first multiple model analysis included covariates and periodontal status. The second model analysis included covariates, PI, GI, and periodontal stage. The third model analysis included covariates, PI, GI, and periodontal grade. Statistical analysis was performed with SPSS for Windows version 22.0 and a p value < 0.05 was accepted as statistically significant.

## Results

### Demographic variables

Table 1 reports the characteristics of the study population. Age was lower in the PH group than in other groups (*p* = 0.001). BMI, sex, smoking, smoking pack years, systemic disease status, and medication used for the systemic disease were not statistically significantly different among the groups. The frequency of dental check-ups was higher in the PH group than in other groups (*p* < 0.001). The frequency of tooth brushing was higher in the IH + PH and PH groups than in the IP + P and P groups (*p* < 0.001).

**Table 1:** Characteristics of the study population

Variables		IP + PH Group (*n* = 35)	IH + PH Group (*n* = 35)	*P* Group (*n* = 35)	PH Group (*n* = 35)	*P* Value
Age (years)		57 (48–61) *,†	49 (42–63)	51 (40–59) *,†	24 (24–29)	0.001
BMI (kg/m ^2^ )		26.12 (23.74–28.48)	25.50 (22.58–28.73)	25.62 (24.57–28.40)	24.39 (21.77–26.56)	0.079
Sex	Male	21 (60)	23 (65.7)	20 (57.1)	19 (54.3)	0.793
	Female	14 (40)	12 (35.3)	15 (42.9)	11 (45.7)	
Frequency of dental check-ups	Every 6 months	1 (2.9) *	6 (17.1)	0 (0)	11 (31.4)	<0.001
	Annually	4 (11.4) *	8 (22.9) *	7 (20) *	21 (60)	
	Irregular	30 (85.7) *	21 (60) *	28 (80) *	3 (8.6)	
Tooth brushing frequency	No	5 (14.3)	1 (2.9)	5 (14.3)	0 (0)	<0.001
	Irregular	3 (8.6) *	2 (5.7) *	12 (34.3) *,†	0 (0)	
	Once a day	18 (51.4) *	10 (28.6)	7 (20)	6 (17.1)	
	More than once a day	9 (25.7) *,†	22 (62.9)	19 (47.5) *,†	29 (82.9)	
Smoking	No	24 (68.6)	25 (71.4)	25 (71.4)	26 (74.3)	0.964
	Yes	11 (31.3)	10 (28.6)	10 (28.6)	9 (25.7)	
Smoking pack years	Never smoked	22 (62.9)	30 (83.3)	24 (68.5)	27 (77.14)	0.42
	≤ 20	8 (22.9)	5 (13.9)	8 (22.9)	7 (20)	
	>20	5 (14.2)	1 (2.8)	3 (8.6)	1 (2.8)	
Systemic disease status	No	23 (66.7)	31 (88.6)	25 (71.4)	29 (82.9)	0.402
	CVD	6 (17.1)	1 (2.9)	3 (8.6)	2 (5.7)	
	Diabetes	5 (14.3)	2 (5.7)	5 (14.3)	2 (5.7)	
	Others	1 (2.9)	1 (2.9)	2 (5.7)	2 (5.7)	
Medication used for systemic disease	No	26 (73.3)	33 (94.3)	29 (82.9)	29 (82.9)	0.161
	Yes	9 (25.7)	2 (5.7)	6 (17.1)	6 (17.1)	

*P* values were obtained from Kruskal Wallis test and Dunn Multiple Comparison test for numerical variables

*P* values were obtained from Chi-square test with Bonferroni correction for categorical variables

Data are expressed as median and 25% to 75% and number (percentage) Statistically significant at *p* < 0.05

^*^ *P* < 0.05 versus IH + PH † *P* < 0.05 versus PH

IP + P: Peri-implantitis and periodontitis IH + PH: Peri-implant and periodontal Health P: periodontitis PH: periodontally healthy BMI: body mass index

### Clinical periodontal findings

Table 2 reports the periodontal conditions of the study groups. Full mouth and tooth sampling site PI, GI, BOP (%), PPD, and CAL were lower in the IH + PH and PH groups than IP + P and P groups (*p* = < 0.001). The number of missing teeth was lower in the PH group than in other groups (*p* = < 0.001). The prevalence of Stage III cases was higher in the P group than in the IP + P group (*p* = 0.041), while Grade B was higher in the IP + P group than P group and grade III was higher in the P group than in the IP + P group (*p* = 0.008). Age and number of implants were higher in the IP + P group than in the IH + PH group.

**Table 2:** Clinical periodontal parameters and GCF volume among all groups

Variable	IP + *P* Group (*n* = 35)	IH + PH Group (*n* = 35)	*P* Group (*n* = 35)	PH Group (*n* = 35)	*p*-value
PI (full mouth)	1.57 (1.25–2) *,†	0.98 (0.3–1.19) *	2.2 (1.71–3.0) *,†	1.04 (0.32–1.30)	< 0.001
GI (full mouth)	2 (1.75–2) *,†	0.47 (0.13–1.08)	2 (1.89–3) *,†	0.79 (0.18–1.08)	< 0.001
BOP (%)(full mouth)	100.0 (73.33–100.0) *,†	6.25 (0–15.93)	100.0 (81.4–100.0) *,†	8.04 (1.79–8.93)	< 0.001
PPD (full mouth)	4.27 (3.86–4.66) *,†	2.22 (1.69–2.73) *	4.04 (3.34–4.72) *,†	1.41 (1.27–1.69)	< 0.001
CAL (full mouth)	4.27 (3.86–5) *,†	2.35 (1.69–2.73) *	4.39 (3.47–4.79) *,†	1.42 (1.27–1.73)	< 0.001
Number of missing teeth	11 (4–17) *	4 (1–12) *	4 (2–7) *	0 (0–1)	<0.001
PI (tooth sampling side) PI (implant sampling side)	2 (1–2) *,† 2 (1–1)	0.5 (0–1) 1 (0–1)	2 (2–3) *,† N/A	0 (0–1) N/A	< 0.001 < 0.001
GI (tooth sampling side) GI (implant sampling side)	2 (2–2) *,† 2 (2–2)	0 (0–1) 0 (0–1)	2 (2–3) *,† N/A	0 (0–1) N/A	< 0.001 < 0.001
BOP (%) (tooth sampling side) BOP (%) (implant sampling side)	100 (100–100) *,† 100 (100–100)	0 (0–1) 0 (0–0)	100 (100–100) *,† N/A	0 (0–0) N/A	< 0.001 < 0.001
PPD (tooth sampling side) PPD (implant sampling side)	5.5 (5–6) *,† 6.50 (6–8)	2 (2–2) 2 (2–3)	7 (5.5–9) *,† N/A	1 (1–1.5) N/A	< 0.001 < 0.001
CAL (tooth sampling side) CAL (implant sampling side)	5.5 (5–7) *,† 6.75 (6–8)	2 (2–2) 2 (2–23)	7.25 (5.5–9.5) *,† N/A	1 (1–1.5) N/A	< 0.001 < 0.001
Periodontal Stage St-I St-II St-III St-IV	2 (5.7) 13 (37.1) 5 (14.3) 15 (42.9)		3 (8.6) 7 (20.0) 15 (42.9) ‡ 10 (28.6)		0.041
Periodontal Grade Garde I Garde II Garde III	11 (31.4) 21 (60.0) 3 (8.6)	N/A N/A N/A	9 (25.7) 12 (34.3) ‡ 14 (40) ‡	N/A N/A N/A	0.008
Implant age	4 (3–6)	3 (2–4)	N/A	N/A	0.020
The number of implants	6 (4–8)	4 (2–7)	N/A	N/A	0.020

*P* values obtained from Mann-Whitney U and Kruskal Wallis test and Dunn Multiple Comparison test for non-parametric variables

Data are expressed as median and 25% to 75% and number (percentage)

Statistically significant at *P* < 0.05

IP + P: Peri-implantitis and periodontitis IH + PH: Peri-implant and periodontal Health P: Periodontitis PH: Periodontally healthy

PI: plaque index GI: gingival index BOP: percentage bleeding on probing PPD: probing pocket depth CAL: clinical attachment level

^*^ *P* < 0.05 versus IH + PH group † *P* < 0.05 versus PH group ^‡^ *P* < 0.05 versus IP + P group

### Microscopic findings

Table 3 reports the prevalence of *E. gingivalis* and *T. tenax*. Both parasites in tooth samples were detected more frequently in the IP + P and P groups than in the IH + PH and PH groups (*p* = < 0.001). The prevalence of *E. gingivalis* and *T. tenax* in peri-implant samples was higher in the IP + P group than in the IH + PH group (*p* = < 0.001 and *p* = 0.001, respectively). There was no difference in both parasites detection between tooth and peri-implant samples in the same mouth in the IP + P and IH + PH groups (*p* ≥ 0.05).

**Table 3:** Comparisons of the detection frequency of *E. gingivalis* and *T. Tenax* between groups and tooth and implant site samples in the same mouth

Variables		IP + *P* Group (*n* = 35)	IH + PH Group (*n* = 35)	*P* Group (*n* = 35)	PH Group (*n* = 35)	*P* Value
*E. gingivalis*						
Tooth samples	Yes	16 (45.7) *,†	1 (2.9)	23 (65.7) *,†	1 (2.9)	<0.001
	No	19 (54.3)	34 (97.1)	12 (34.3)	34 (97.1)	
Implant samples	Yes	15 (42.9)	2 (5.7)	N/A	N/A	<0.001
	No	20 (57.1)	33 (94.3)	N/A	N/A	
*P* Value		0.267	0.572			
*T. tenax*						
Tooth samples	Yes	15 (42.9) *,†	3 (8.6)	17 (48.6) *,†	2 (94.3)	
	No	20 (57.1)	32 (91.4)	18 (51.4)	33 (5.7)	< 0.001
Implant samples	Yes	12 (65.7)	1 (2.9)	N/A	N/A	
	No	23 (34.3)	34 (97.1)	N/A	N/A	0.001
*P* Value		0.455	0.289			

*P* values were obtained from Chi-square test with Bonferroni correction

Data were expressed as numbers (percentage) Statistically significant at *p* < 0.05

IP + P: Peri-implantitis and periodontitis IH + PH: Peri-implant and periodontal Health P: periodontitis PH: periodontally healthy

*P* < 0.05 versus IH + PH group † *P* < 0.05 versus PH group ^‡^ *P* < 0.05 versus PH + P group

### Logistic regression models

Table 4 reports results of the univariate and multiple logistic regression model analyses. Three different multiple model analyses were performed to ascertain the relationship between the presence of *E. gingivalis* and other factors. In the first multiple final (third step) model analysis, detection of *E. gingivalis* was increased in both the IP + P group and P group (respectively OR = 36.16; 95% CI: 4.87–289.71; *P* = 0.001 and OR = 79.23; 95% CI: 9.39–668.1; P = < 0.001) compared to the PH group. In the second multiple final (third step) model analysis, PI (OR = 1.81; 95% CI: 0.04–3.16; *P* = 0.036) had a higher risk for detection of *E. gingivalis*. *E. gingivalis* detection was more likely in stage I-II and stage III-IV periodontitis (respectively OR = 9.67; 95% CI: 2.58–36.28; *P* = 0.001 and OR = 24.11; 95% CI: 6.49–89.51; P = < 0.001). In the third multiple final (fourth step) model analysis, periodontitis grade A, B and C were associated with increased risk of detection of *E. gingivalis* compared with periodontal health (respectively OR = 9.34; 95% CI: 2.32–35.15; *P* = 0.001, OR = 23.51; 95% CI: 6.31–87.62; P = < 0.001 and OR = 16.41; 95% CI: 3.18–84.57; P = < 0.001).

**Table 4:** Logistic regression models for the detection frequency of *T.tenax and E.gingivalis* in all samples *(140 patients*,* 210 samples)*

		Univariate Model		Multivariate Final Model *		Multivariate Final Model **		Multivariate Final Model ***	
		OR(95% CI)	*p*	OR(95%-CI)	*p*	OR(95%-CI)	*p*	OR(95%-CI)	*p*
*E. gingivalis*									
Smoking	**Yes** **No**	0.44(0.21–0.91) 1(Reference)	0.027 N/A	0.28(0.12–0.66) 1(Reference)	0.004 N/A	0.3(0.12–0.74) 1(Reference)	0.009	0.27(0.11–0.68) 1(Reference)	0.001 N/A
Frequency of Tooth Brushing	**Regularly** **Irregularly**	0.32(0.16–0.67) 1(Reference)	0.002 N/A	- -	- -	-	-		
Frequency of Dental Check-ups	**Regularly** **Irregularly**	0.46(0.24–0.89) 1(Reference)	0.022 N/A	- -	- -	-	-		
Periodontal Groups	**IP + P** **IH + PH** **P** **PH**	32.11(4.16–247.71) 1.52(0.15–15.19) 65.17(7.92–536.17) 1(Reference)	0.001 0.72 < 0.001 N/A	38.16(4.87–289.71) 1.57(0.16–15.73) 79.23(9.39–668.1) 1(Reference)	0.001 0.703 < 0.001 N/A				
PI (sampling site)		4.31(2.74–6.76)	< 0.001			1.81(1.04–3.16)	0.036	1.93(1.09–3.43)	0.024
GI (sampling site)		4.62(2.85–7.51)	< 0.001			-	-	-	**-**
Periodontitis stage	**Modarete Periodontitis** **(Stage I-II)** **Severe Periodontitis (Stage III-IV)** **Non-Periodontitis**	19.05(5.78–62.76) 40.63(13.24–124.67) 1(Reference)	< 0.001 < 0.001 N/A			9.67(2.58–36.28) 24.11(6.49–89.51) 1(Reference)	0.001 < 0.001 N/A		
Periodontitis Grade	**Garde A** **Grade B** **Grade C** **Non-Periodontitis**	18.24(5.34–62.25) 36.73(11.78–114.51) 37.88 (9.91–144.81) 1(Reference)	0.001 < 0.001 < 0.001 N/A					9.034(2.32–35.15) 23.51(6.31–87.62) 16.41 (3.18–84.57) 1(Reference)	0.001 < 0.001 0.001 N/A
*T. tenax*									
Periodontal Groups	**IP + P** **IH + PH** **P** **PH**	10.36(2.29–46.72) 1(0.17–5.74) 15.58(3.23–75.18) 1(Reference)	0.002 1 0.001 N/A	**-** **-** **-** **-**	**-**				
PI (sampling site)		2.72(1.42–3.01)	< 0.001			**-**	-	-	**-**
GI (sampling site)		2.88(1.91–4.36)	< 0.001			**-**	-	-	**-**
Periodontitis Stage	**Modarete Periodontitis** **(Stage I-II)** **Severe Periodontitis (Stage III-IV)** **Non-Periodontitis**	11.14(3.88–31.94) 12.25(4.67–32.11) 1(Reference)	< 0.001 < 0.001 N/A			9.43(3.28–27.1) 11.79(4.51–30.88) 1(Reference)	< 0.001 < 0.001 N/A	-	
Periodontitis Grade	**Garde A** **Grade B** **Grade C** **Non-Periodontitis**	6.75(2.18–20.93) 15.32(5.74–40.89) 13.5(4.04–45.1) 1(Reference)	0.001 < 0.001 < 0.001 N/A					6.61(2.13–20.51) 13.28(4.93–35.81) 13.23(3.96–44.21) 1(Reference)	0.001 < 0.001 < 0.001 N/A

Statistically significant at *P* < 0.05

IP + P: Peri-implantitis and periodontitis IH + PH: Peri-implant and periodontal Health P: periodontitis PH: periodontally healthy

BMI: body mass index PI: plaque index GI: gingival index

*independent factors: age, sex, BMI, smoking status, systemic health status, frequency of tooth brushing, frequency of dental check-ups, periodontal status

**independent factors: age, sex, BMI, smoking status, systemic health status, frequency of tooth brushing, frequency of dental check-ups, PI, GI, periodontal stage

*** independent factors: age, sex, BMI, smoking status, systemic health status, frequency of tooth brushing, frequency of dental check-ups, PI, GI, periodontal grade

Two different multiple model analyses were performed to ascertain the relationship between the presence of *T.tenax* and other factors. In the first multiple final (third step) model analysis, having periodontitis stage I-II and stage III-IV were associated with detection of *T. tenax* compared to non-periodontitis (respectively OR = 9.43; 95% CI: 3.28–27.1; *P* = < 0.001 and OR = 11.79; 95% CI: 4.51–30.88; *P* = < 0.001). In the second multiple final (fourth step) model analysis, detection of *T. tenax* was associated with Grade A periodontitis (OR = 6.61; 95% CI: 2.13–20.51; *P* = 0.001), Grade B periodontitis (OR = 13.28; 95% CI: 4.93–35.81; *P* = < 0.001) and Grade C periodontitis (OR = 13.23; 95% CI: 3.96–44.21; P = < 0.001) compared to periodontal health.

Model analysis was performed to ascertain the association between the presence of parasites and implant age and implant numbers in implant samples (IP + P and IH + PH groups = 70 samples). The presence of *T. tenax* and *E. gingivalis* was not associated with the implant and implant numbers. (Supplementary Table 1).

## Discussion

The association of two potentially pathogenic protozoans with peri-implantitis and periodontitis lesions was examined. The detection frequency of E. gingivalis and T. tenax was evaluated in peri-implantitis and periodontitis lesions, both across and within individuals with teeth and implants. The findings of the present study show that the detection frequency *of E. gingivalis* and *T. tenax* parasites was higher in peri-implantitis and periodontitis lesions compared to healthy sides. Both parasites had a similar prevalence on peri-implant and tooth surfaces. *E. gingivalis* and *T. tenax* had an association between the presence of peri-implantitis and periodontitis lesions, periodontal disease severity, and grading.

The research regarding the restoration of lost teeth via dental implants in patients with a history of periodontitis has mainly focused on inflammatory markers and bacterial species [37, 38]. However, protozoan parasites have rarely been investigated [13, 39, 40]. The previous studies showed that the prevalence of *E. gingivalis* and *T. tenax* were higher in patients with periodontitis than in controls [13, 39]. Another study showed that *E. gingivalis* and *T. tenax* decreased in saliva and plaque samples after non-surgical periodontal treatment [40]. The present study included two different groups (IP + P and P groups) for periodontitis samples. The present findings represent detection frequencies based on microscopic identification and do not provide quantitative data regarding parasite levels. Compatible with the previous studies, the detection frequency of *E. gingivalis* and *T. tenax* was higher in these groups compared to the periodontal healthy groups (IH + PH and PG groups). A previous study [41] found that *T. tenax* and *E. gingivalis* were present in 33.6% and 30.7% of peri-implantitis lesions, respectively, while absent in healthy implant sites. Similar associations were confirmed by this study, and higher detection of *E. gingivalis* (42.9%) and *T. tenax* (65.7%) in the IP group compared to the IH group, confirming the presence of these protozoans in peri-implantitis. Also, in multivariate model analysis, the relative risk increased with the PI + P and P group with 38.16- and 79.23-fold risk with the association of *E. gingivalis* independently of other factors. *T. tenax* showed the same risk association for the presence of diseases in the univariate model analysis. Multilevel analysis was not performed for *T. tenax* since other factors did not show significant associations in univariate analysis. The fact that E. gingivalis and T. tenax colonize and are found in larger numbers in inflamed gingival sulcus and periodontal pockets does not necessarily imply a direct pathogenic role, as they may potentially have indirect effects on influencing dysbiosis [14]. Strong modulation of bacterial genes in periodontitis lesions can promote a dysbiotic environment by both influencing and being influenced by E. gingivalis parasites [16]. E. gingivalis is known to inhibit host cell proliferation through direct contact, cytoplasmic penetration, and feeding on the chromatin of human gingival epithelial cells, and unlike P. gingivalis, it strongly increases the expression of interleukin-8 and the epithelial barrier gene MUC21 in epithelial cells, and also increases the expression of collagenase matrix metallopeptidase-13 in gingival fibroblasts [15]. It has been shown that T. tenax trophozoites can stimulate the release of pro-inflammatory cytokines, including IL-8, macrophage migration inhibitory factor (MIF), IL-1, intercellular adhesion molecule-1 (ICAM-1), and IL-1 receptor antagonist (IL-1ra), which play a role in neutrophil recruitment, bone resorption, macrophage activation, and vascular permeability [42]. It can be suggested that oral protozoans may potentially play a role in the feedback loop mechanism between the dysbiotic community and the host immune response, which are involved in the pathogenesis of periodontal disease [14]. Also, the higher detection frequency of *E. gingivalis* and *T. tenax* in peri-implantitis compared to peri-implant health aligns with the hypothesis that these protozoa may contribute to the pathogenesis of peri-implant disease through similar mechanisms as in periodontitis.

Teeth and implants shared a small abundance of bacterial species, and peri-implantitis lesions have less bacterial diversity compared to periodontitis [43]. It has been suggested that the peri-implant microbiota is distinct and that a new model for peri-implant infections is required [44]. The present study findings showed that the detection frequency of *E. gingivalis* and *T. tenax* was similar in peri-implantitis and periodontitis in the same host. The results of the present study may be important to evaluate the diversity of periodontal and peri-implant microbial communities. The similar prevalence of oral parasites in both teeth and implants may indicate that parasites are one of the core microbial species in both lesions.

In logistic regression models, while PI was associated with a higher risk of *E. gingivalis* detection, smoking was associated with a lower risk, which contrasts with its well-established role as a risk factor for periodontal disease [45]. Because PI is considered the major etiological factor of periodontitis presence and severity, the positive correlation of *E. gingivalis* with increased bacterial plaque mass may be another indicator explaining its relationship with periodontal disease [45]. On the other hand, possibly due to tobacco exerting deleterious effects on the nondetected species, smoking can have a negative effect on the prevalence of *E. gingivalis* [46].

The microbial community varies according to the severity of periodontal disease [47]. The previous studies indicated that *E. gingivalis* and *T. tenax* have a link between periodontitis severity and extent [13, 39]. Compatible with previous studies [13, 39], the relative risk increased with the periodontitis severity and grading, with the association of *E. gingivalis* independently of other factors in the present study. In parallel, the relative risk increased with the association of *T. tenax.* These findings indicate the potential role of oral protozoa in the severity and progression of periodontal disease. Another interesting aspect to consider is the effectiveness of the use of systemic metronidazole in peri-implantitis treatment [48, 49]. Metronidazole is one of the main drugs used against anaerobic bacteria and protozoa, such as *Trichomonas vaginalis*, *Entamoeba histolytica*, and *Giardia lamblia* [50]. It could be speculated that the success of metronidazole in the treatment of peri-implantitis and periodontitis could potentially be attributed to its efficacy against two major oral protozoa, which are *E. gingivalis* and *T. tenax*.

The study employed robust methodologies, including standardized clinical examinations, well-defined inclusion and exclusion criteria, and blinded microscopic assessments, which enhanced the reliability of the findings. The use of multilevel logistic regression analyses allowed for the adjustment of potential confounders, providing a comprehensive evaluation of the associations between parasite presence and clinical parameters. However, several limitations must be acknowledged. The cross-sectional design impedes causal inferences regarding the role of *E. gingivalis* and *T. tenax* in the pathogenesis of periodontal and peri-implant diseases. Longitudinal studies are needed to establish sequential relationships. Also, a group with peri-implantitis and periodontally healthy individuals would have provided additional biological insight. However, due to the recruitment characteristics of the study population, this subgroup could not be represented. Additionally, no data are available about any periodontal treatment > 6 months before baseline. While the microscopic methods used were effective for parasite detection, advanced molecular techniques such as polymerase chain reaction (PCR) could provide more sensitive and specific results. One limitation of the present study is that the microscopic detection of the tested parasites was not verified using PCR-based molecular analysis. Investigations regarding how these parasitic protozoa interact with host immune responses and the oral microbiome can reveal important information about their involvement in periodontal and peri-implant disease progression. Investigating the possibility of utilizing these protozoa as therapeutic targets in peri-implant and periodontal disease management also requires further research.

## Conclusion

Within the limitations of this study, the findings suggested that *T. tenax* and *E. gingivalis* may be associated with peri-implantitis and periodontitis lesions. The detection of parasites is similar on tooth and implant surfaces in the same mouth. Severity and grading of periodontitis were associated with *E. gingivalis* and *T. tenax*. Protozoa found at sites of disease may not directly cause these conditions but their detection serves as a warning for their potential involvement as opportunistic pathogens or indicators of oral inflammation. This research adds to existing knowledge about the dysbiosis of periodontal and peri-implant diseases while establishing a foundation for upcoming studies on diagnostic and treatment approaches.

## Supplementary Information

Below is the link to the electronic supplementary material.

Supplementary File 1 (DOCX 132 KB)

Supplementary File 2 (DOCX 16.9 KB)

## Data Availability

All data sets used in this study are not publicly available due to ethical or legal restrictions. For inquiries regarding the data sets used in this study, please contact Aysegul Sari, the principal investigator of this study, with reasonable requests.
