# Supplementary material for: The association between Trichomonas tenax and Entamoeba gingivalis and periimplantitis and periodontitis
Source: Clin Oral Investig. 2026 Jul 17;30(8):343. doi: 10.1007/s00784-026-07036-x (PMC13379453; doi:10.1007/s00784-026-07036-x)
Supplement: Supplementary file 1 — (DOCX 132 KB) [file 784_2026_7036_MOESM1_ESM.docx]

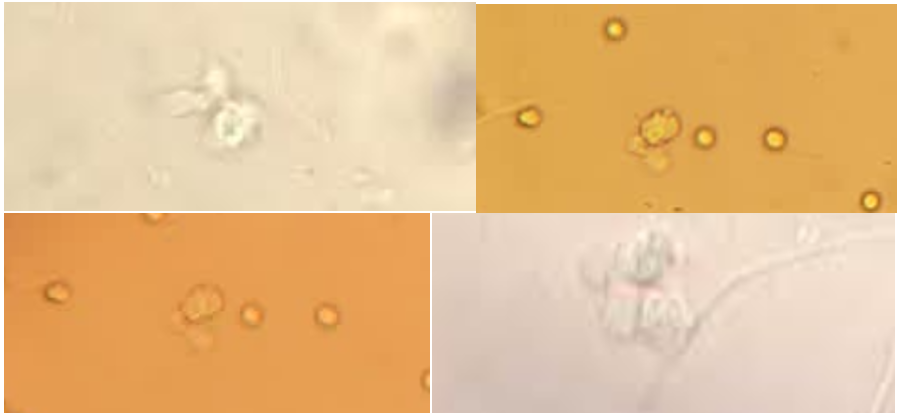


**Supplementary Figure 1.** Representative microscopic images showing *Entamoeba gingivalis* detected in the examined samples.
