# Supplementary material for: The association between Trichomonas tenax and Entamoeba gingivalis and periimplantitis and periodontitis
Source: Clin Oral Investig. 2026 Jul 17;30(8):343. doi: 10.1007/s00784-026-07036-x (PMC13379453; doi:10.1007/s00784-026-07036-x)
Supplement: Supplementary file 2 — (DOCX 16.9 KB) [file 784_2026_7036_MOESM2_ESM.docx]

**Supplementary Table 1 |** Logistic regression models for the presence of *T.tenax and E.gingivalis* in implant samples (*70 patients*)

|  |  | **Univariate Model** |  |
| --- | --- | --- | --- |
|  |  | **OR (*95% CI)*** | **p** |
| ***T. tenax*** |  |  |  |
| **Implant Age** |  | 1.032 (0.83-1.29) | 0.779 |
| **Implant Number** |  | 1.19 (0.99-1.42) | 0.067 |
| ***E. gingivalis*** |  |  |  |
| **Implant Age** |  | 1.19 (0.98-1.44) | 0.088 |
| **Implant Number** |  | 1.09 (0.92-1.28) | 0.327 |

*The models adjusted for age, sex, BMI, smoking status, systemic health status, frequency of tooth brushing, frequency of dental check-ups
